# Surviving the Storm: The Role of Poly‐ and Depolyploidization in Tissues and Tumors

**DOI:** 10.1002/advs.202306318

**Published:** 2024-04-17

**Authors:** Yucui Zhao, Sijia He, Minghui Zhao, Qian Huang

**Affiliations:** ^1^ Cancer Center Shanghai General Hospital Shanghai Jiao Tong University School of Medicine Shanghai 201620 China; ^2^ Department of Radiation Oncology Second Affiliated Hospital Zhejiang University School of Medicine Hangzhou 310009 China; ^3^ Department of Radiation Oncology First Affiliated Hospital of Nanjing Medical University Nanjing 210029 China; ^4^ Shanghai Key Laboratory of Pancreatic Diseases Shanghai General Hospital Shanghai Jiao Tong University School of Medicine Shanghai 201620 China

**Keywords:** cancer resistance, cytotoxic therapy, depolyploidization, polyaneuploid giant cancer cells, polyploidization

## Abstract

Polyploidization and depolyploidization are critical processes in the normal development and tissue homeostasis of diploid organisms. Recent investigations have revealed that polyaneuploid cancer cells (PACCs) exploit this ploidy variation as a survival strategy against anticancer treatment and for the repopulation of tumors. Unscheduled polyploidization and chromosomal instability in PACCs enhance malignancy and treatment resistance. However, their inability to undergo mitosis causes catastrophic cellular death in most PACCs. Adaptive ploid reversal mechanisms, such as multipolar mitosis, centrosome clustering, meiosis‐like division, and amitosis, counteract this lethal outcome and drive cancer relapse. The purpose of this work is to focus on PACCs induced by cytotoxic therapy, highlighting the latest discoveries in ploidy dynamics in physiological and pathological contexts. Specifically, by emphasizing the role of “poly‐depolyploidization” in tumor progression, the aim is to identify novel therapeutic targets or paradigms for combating diseases associated with aberrant ploidies.

## Graphical abstract

This work delves into the intricate dynamics of ploidy variation in both normal tissues and cancer cells. It further discusses the survival and propagating strategy employed by cancer cells, as they undergo polyploidization to withstand the storm of cytotoxic oncology, and depolyploidization to drive tumor repopulation. Finally, it proposes novel therapeutic targets for combating cancer.

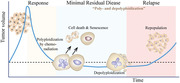

## 1 Introduction

Diploidy (2N), characterized as having two complete sets of homologous chromosomes, is the typical condition for sexually reproducing eukaryotic organisms. While polyploidy (>2N, with one or more additional sets of chromosomes) is common in nature, mammals are unable to undergo germline polyploidization. Accumulating evidence suggests that ploidy variation and heterogeneity are vital mechanisms diploid organisms employ to confront adverse conditions while maintaining homeostasis. Besides polyploidy, variations can also manifest as an abnormal chromosome number (aneuploidy) or regional ploidy changes. Hepatocytes exemplify a wide spectrum of genomic content, with polyploidy observed in ≈50% of cells and aneuploidy affecting 30–90% of human hepatocytes.^[^ ^1^ ^]^ Specifically, ≈5% of polyploid hepatocytes are programmed for polyploidy reversal, generating aneuploid cells.^[^ ^2^ ^]^ This phenomenon of aneuploidy is postulated to function as a genomic diversity buffer, fostering the adaptability and resilience of the liver in the face of various challenges.

These ploidy dynamics are also prevalent in human cancers, correlating with therapeutic resistance and disease progression. Aneuploidy occurs in nearly 90% of solid tumors,^[^ ^3^ ^]^ with chromosome arm‐level changes predicting the response to chemotherapeutic drugs.^[^ ^4^ ^]^ Chromosomal instability (CIN) drives aneuploidy by increasing the frequency of missegregation events during mitosis. This instability accelerates resistance to cytotoxic therapies by enhancing karyotype heterogeneity, leading to further clonal selection and expansion.^[^ ^5^ ^]^ Polyploidization, or whole‐genome doubling (WGD), occurs in one‐third of human cancers^[^ ^6^ ^]^ and over half of metastatic cancers.^[^ ^7^ ^]^ Tumors that undergo polyploidization frequently display aneuploidy due to elevated CIN levels.^[^ ^8^ ^]^ Polyploid cells are more tolerant to chromosomal imbalances associated with aneuploidy, which are strongly correlated with treatment resistance and poor prognosis in diverse cancers.^[^ ^6^, ^9^ ^]^

**Figure 1:**
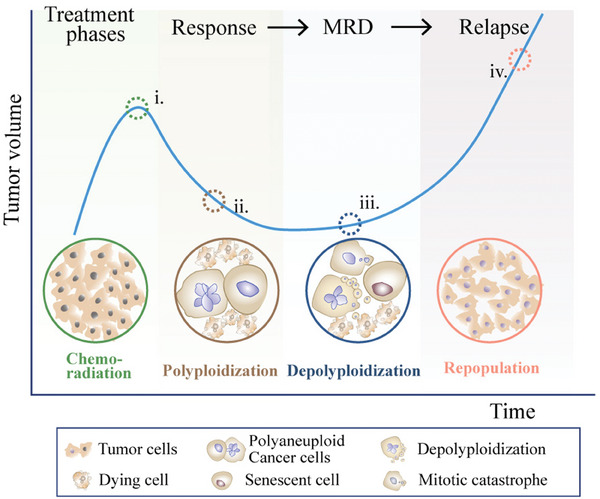
The role of polyaneuploid giant cancer cells (PACCs) in cancer relapse. PACCs, characterized by an extremely complex karyotype, were previously thought to be a “dead end” owing to their inability to undergo mitosis, leading to the assumption that they function as a tumor‐suppressive mechanism. However, emerging evidence challenges this notion, suggesting that PACCs serve a more dynamic role. At the macroscopic level, despite the initial cancer remission following cytotoxic therapy, eventual repopulation, and relapse occur after a dormant state known as minimal residual disease (MRD). At the microscopic level, in response to cytotoxic anticancer treatment (i, green circles), the majority of surviving para‐diploid cancer cells undergo polyploidization, generating mono‐ or multinucleated PACCs (ii, maroon circles). During the transitional state (iii, blue circles), most PACCs commit mitotic catastrophe or senescence, leading to massive cell death. However, a small fraction of PACCs manage to survive and repopulate the tumor (iv, pink circles), giving rise to small, para‐diploid clonal progeny with increased resistance.

Longitudinal imaging of therapy‐resistant cancer cells revealed a subset of giant cancer cells with enlarged mono‐/multiple nuclei. Polyaneuploid cancer cells (PACCs), which are aneuploid cancer cells that have undergone polyploidization,^[^ ^10^ ^]^ have been observed in vitro and in vivo across multiple cancer types following cytotoxic chemoradiation.^[^ ^11^ ^]^ PACCs have previously been speculated to be a dead end following mitotic catastrophe or persistent dormancy. However, this contradicts the fact that the occurrence of PACCs increases with malignancy grade and is associated with poor prognosis.^[^ ^6^, ^11^, ^12^ ^]^ Emerging evidence has shown that not all PACCs die down inevitably; instead, this state strongly corresponds to the clinical concept of minimal residual disease (MRD), representing the stage of maximal tumor shrinkage prior to subsequent tumor progression (**Figure** **1**). Our recent study found that a fraction of PACCs can survive radiation and enter a state of radiation‐tolerant persistence, remaining dormant for extended periods.^[^ ^11^, ^13^ ^]^ These cells, referred to as radiation‐tolerant persister cells, function similarly to persister cells that arise after targeted drug therapy.^[^ ^14^ ^]^ Furthermore, they can escape from the slow‐cycling state and generate new tumor cells similar to the parent cells through viral budding‐like division. Similar phenomena have been observed in other studies investigating cancer resistance to chemotherapy, whereby cancer cells formed PACCs and underwent asymmetric cell division, giving rise to regular‐sized diploid daughter cells.^[^ ^11^, ^15^ ^]^ These newborn cells possess enhanced survival signaling, invasion ability, and resistance to chemotherapy,^[^ ^16^ ^]^ leading to the failure of anticancer treatments.

Preliminary efforts have been made to identify these morphologically distinct cancer cells; however, the lack of a unified nomenclature remains a major obstacle. These cancer cells have different terms, including polyploid or polymorphous giant cancer cells, multinucleated cancer cells, and osteoblast‐like/blastocyst cancer cells.^[^ ^11a^ ^]^ Herein, we distinguished PACCs from non‐proliferative polyploid normal cells during the differentiation of specialized tissues.^[^ ^17^ ^]^ In this review, by analogy with cases of polyploidy in normal development and response to injury or damage, we summarize polyploidization events in tumor progression following anticancer treatment. We also synthesize current insights and discuss the possible modes of depolyploidization, further highlighting the therapeutic value of PACCs in overcoming tumor progression.

## 2 Advantages and Origins of Polyploidization in Normal Tissue and Tumor Evolution

### 2.1 Why Does Polyploidization Matter?

Polyploidization is commonly observed in various taxa, particularly fungi, plants, and insects. By reshaping genetic and physiological characteristics, polyploidization enables functional adaption and enhances resilience against pathological challenges. In the context of physical barriers, an obvious advantage of polyploid cells is their large size. A notable example is the mono‐ and multinucleated polyploid subperineurial glia, which are essential components of the *Drosophila* blood–brain barrier.^[^ ^18^ ^]^ Additionally, increased gene expression and protein production in polyploid cells may be conducive to a higher growth rate in early maturation and faster tissue repair during wound healing.^[^ ^19^ ^]^ Further, polyploidization serves as an alternative strategy for stem cell maintenance in the *Drosophila* gut epithelium. Under normal physiological conditions, the InR/Pi3K/TOR pathways regulate stem cell division and enterocyte polyploidization during homeostasis. During severe damage, EGFR/MAPK signaling is boosted to increase ploidy levels in enterocytes to compensate for restricted stem cell division.^[^ ^20^ ^]^ Furthermore, because polyploidization often leads to enhanced genetic diversity, it may protect cells from genotoxic damage‐induced mutations.^[^ ^21^ ^]^

In humans, somatic polyploidy of varying degrees is tolerated owing to its programmed roles in normal development (such as the placenta) or stress responses (e.g., liver or kidney injury).^[^ ^21^, ^22^ ^]^ However, a high degree of polyploidy can be detrimental to fitness because of CIN, interrupted cell division (including mitotic and meiotic processes), and disruptive changes in gene expression and epigenetic regulation.^[^ ^23^ ^]^ For example, the polyploidization of tubular cells serves as a rapid recovery mechanism after acute renal injury, whereas sustained polyploidization leads to progressive fibrosis and chronic kidney disease.^[^ ^24^ ^]^

Unprogrammed WGD is a prominent characteristic of the cancer genomes. Cells that undergo successive WGD events exhibit polyploidy with genomic instability and rapidly accumulate numerical and structural chromosomal abnormalities. This phenomenon contributes to increased tumor cell diversity and accelerates cancer genome evolution with adaptive potential, especially when subjected to genotoxic therapies.^[^ ^25^ ^]^ In contrast, polyploid cancer cells possess a greater capacity to survive against the long‐lasting negative effects of CIN than their diploid counterparts.^[^ ^26^ ^]^ The noteworthy impact of polyploidization lies in countering the lethal effects of “Müller's ratchet” in asexual reproduction, where harmful mutations accumulate irreversibly over time due to lack of recombination.^[^ ^9a^ ^]^ Cancer cells also experience an irreversible accumulation of deleterious passenger genes, particularly in genomic regions with loss of heterozygosity (LOH). By providing additional mutation‐free segments, polyploidization is preferentially selected as a mechanism to alleviate the ratchet‐like effect of somatic mutations in LOH regions.^[^ ^27^ ^]^ A recent study provided evidence supporting the involvement of polyploidization in tumor evolution, transitioning from the precancerous state after sporadic p53 inactivation. Utilizing a lineage‐traced mouse model, that study unequivocally showed that polyploidization, in a deterministic manner, emerged as a mechanism to mitigate significant chromosomal losses accrued following the loss of p53 and then acquiring further gains and amplifications, thus fueling tumor progression.^[^ ^28^ ^]^ By generating and buffering aneuploidy, polyploidization can contribute to further adaptive events, such as therapeutic resistance.^[^ ^29^ ^]^

### 2.2 How Does Polyploidization Occur?

**Figure 2:**
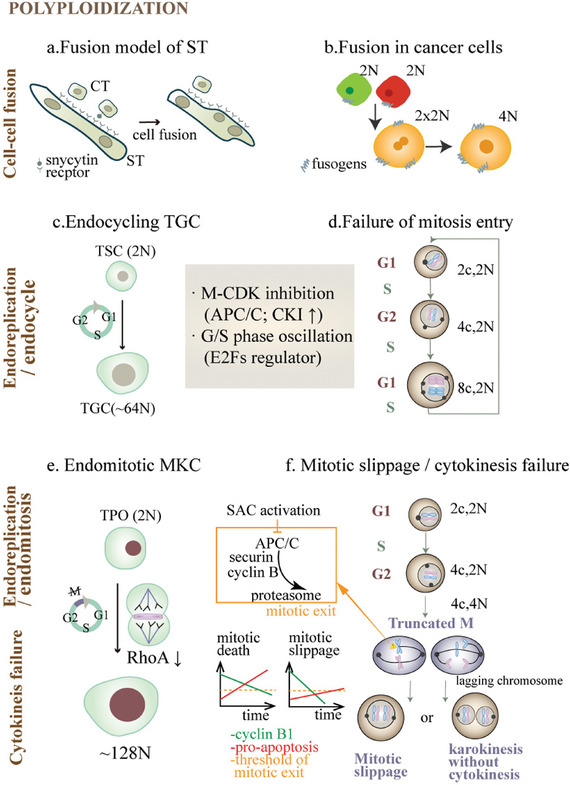
Examples and mechanisms of polyploidization during normal and oncogenic development. Polyploid cells can arise through diverse pathways, including cell‐cell fusion, endoreplication, and cytokinesis failure. Endoreplication involves endocycle and endomitosis. Panel a) is the fusion model of syncytiotrophoblasts (ST) in rodent placenta, where attachment of syncytiotrophins to their receptors triggers the fusion of cytotrophoblasts into ST. Panel b) depicts cell‐cell fusion in the formation of PACCs. Evidence suggests that PACCs can exhibit hybrid fluorescence originating from two different types of fluorescence in two cancer cells. The endocycle represents an aberrant cell cycle comprising alternating G and S phases, achieved by a combination of M‐phase cyclin‐dependent kinase (M‐CDK) inhibition and G/S phase oscillation. Panel c) showcases an example of an endocycling trophoblast giant cell (TGC) involved in mouse embryo development. TGC differentiate from trophoblast stem cells (TSC) through the regulation of E2Fs and CKI. Panel d) demonstrates an endocycling cancer cell that undergoes endoreplication without mitosis owing to dysregulation of cyclin‐CDK activity. Cells undergoing endomitosis initiate mitosis but prematurely exit, resulting from defects in spindle checkpoints or cytokinesis regulators. Panel e) illustrates the regulation of thrombopoietin (TPO) in the generation of polyploid megakaryocytes (MKCs), which undergo rounds of endomitosis to achieve a sufficient size for budding off anucleated platelets. Defects in RhoA activity impair the formation of the actomyosin ring during abscission, leading to cytokinesis failure. Panel f) shows the process of mitotic slippage and cytokinesis failure in cancer cells. These cells undergo mitotic arrest with prolonged spindle assembly checkpoint (SAC) activation during metaphase, accompanied by gradual degradation of cyclin B1 and repression of pro‐apoptotic signals. Rapid degradation of cyclin B1 and delayed induction of pro‐apoptotic signals during mitotic arrest can result in mitotic slippage, thereby producing a mononucleated PACC. Cytokinesis failure can occur due to any obstacles emerging during cytokinesis, resulting in the formation of multinucleated PACCs. Abbreviations: APC/C, anaphase‐promoting complex/cyclosome; CKI, cyclin‐dependent kinase inhibitor; CT, cytotrophoblasts; MKC, megakaryocyte; SAC, spindle assembly checkpoint; ST, syncytiotrophoblasts; TGC, trophoblast giant cell; TPO, thrombopoietin; TSC, trophoblast stem cell.

Several routes can lead to cells switching from a diploid to a polyploid state, including cell‐cell fusion, endoreplication, and cytokinesis failure (**Figure** **2** and **Table** **1**).

**Table 1:** Recent studies on PACCs elucidate mechanisms of poly‐ and depolyploidization using in vitro and in vivo experimental cancer models.

Stimulus	Tumor type	Model system	Mode/Mechanism of Polyploidization	Mode/Mechanism of Depolyploidization	References
Spontaneous	High‐grade serous carcinoma	MDA‐HGSC‐2414 organoid	Nuclear fragmentation or fusion, endocycling, endomitosis/increased nuclear‐to‐cytoplasmic ratio similar to pre‐embryogenesis	Amitosis‐nuclear budding/decreased nuclear‐to‐cytoplasmic ratio mimicking blastomere's division	[11d]
Radiation	Colorectal cancer	HCT116, Xenografts, PDX	–	Amitosis‐viral budding‐like division/Suppressed type‐I interferon‐mediated antiviral signaling	[13]
PARP inhibitor (olaparib)	Ovarian and breast cancer	Hey, organoid, PDX	Endoreplication / de‐repressing the embryonic program which can be blocked by antiprogestin contraceptive drug mifepristone	Bipolar or tripolar mitosis	[15a]
hypoxia‐mimic CoCl_2_, ‐	Colorectal, breast cancer	Multiple cell lines	Spontaneous cell fusion with novel oncogenic features/fusion‐related proteins involved	Partial ploidy reduction with genome recombination and phenotypic diversification	[35, 38]
Cyclin E1 overexpression	Hepatocellular cancer, osteosarcoma	Transgenic mice, U2OS	Endocycling/precocious‐S phase entry, driving p53‐p21 mediated mitotic bypass; suppressed p53‐dependent senescence	‐	[43, 44]
Endocytic genes mutation	Larval wing imaginal disc	Drosophila tumor model	Endoreplication/ JNK and Yorkie (Yap homolog)‐mediated downregulation of cyclin B	–	[51]
inhibitors of actin polymerization (cytochalasin D or B), myosin II inhibitor (blebbistatin)	Breast cancer and others	MCF10A, RPE‐1	Cytokinesis failure	Clustering extra centrosomes to a bipolar division/regulation of microtubule motors (HSET, cortical dynein), genetic mutation (PP2A‐Aα loss of function)	[73, 75, 76]
Radiation, etoposide, doxorubicin, paclitaxel	Lymphoma, ovarian teratocarcinoma, breast cancer	Ramos, Namalwa, PA1,HeLa	–	Meiosis‐like reductive division likely with recombination events	[77c]
Notch‐ induction	Salivary gland imaginal ring transition‐zone cancer	Drosophila tumor model	Endoreplication/upregulation of the Stg (Cdc25 homolog).	Meiosis‐like reductive division/DNA‐damage response and repair genes are required	[78]

### 2.3 Cell‐Cell Fusion

Cell fusion is commonly observed in myoblasts that form muscle fibers,^[^ ^30^ ^]^ macrophages during bone remodeling,^[^ ^31^ ^]^ and cytotrophoblasts (CTs) that contribute to placental formation.^[^ ^32^ ^]^ This process is important in tissue development and functional reconstruction. Defects in cell fusion can result in congenital myopathy, osteopetrosis, and preeclampsia.^[^ ^33^ ^]^ During cell fusion, specialized proteins known as fusogens are required to overcome energy barriers and facilitate membrane fusion. In the well‐studied placental syncytium (Figure 2a), polyploid syncytiotrophoblasts (ST) are continuously formed through the fusion of mononucleated CTs and act as the primary feto‐maternal interface. Proteins called syncytins (types 1 and 2), which are of endogenous retroviral origin, interact with their respective receptors (ASCT1 and ASCT2) to initiate the fusion of CTs into ST.^[^ ^34^ ^]^

Cell fusion also plays a key role in the formation of polyaneuploidy in tumors (Figure 2b). Zhang et al. observed that 10–20% of PACCs in breast and ovarian cell lines could be formed via cell fusion under CoCl_2_‐mediated hypoxia.^[^ ^16b^ ^]^ Fusion‐related proteins, notably syncytin‐1, CD9, and CD47, are involved in this process, validating the functional role of cell fusion in PACC generation.^[^ ^35^ ^]^ Another study identified that radiation‐induced PACCs are generated from cell fusion at a frequency of 50–70%; these cells escape lethal doses of radiation and eventually lead to tumor repopulation.^[^ ^36^ ^]^ Following cell fusion, hybrid cancer cells demonstrate amplified oncogenic characteristics during oncogenesis and metastasis,^[^ ^37^ ^]^ producing diverse progenies via parasexual recombination and partial ploidy reduction, further facilitating evolutionary adaptation to selective pressures.^[^ ^38^ ^]^

### 2.4 Endoreplication

As an alternative cell cycle progression, cells in endoreplication replicate their genomes successively without completing cytokinesis in the mitosis (M) phase. There are two variants of endoreplication: the endocycle and endomitosis.^[^ ^39^ ^]^ In an endocycling cell,^[^ ^40^ ^]^ mitosis is completely bypassed; instead, the cell alternates between the synthesis (S) and gap (G) phases, generating a mononucleated PACC. In contrast, endomitotic cells initiate mitosis but undergo an incomplete, truncated form of the M phase, exiting either the nuclear envelope breakdown phase, nuclear division phase, or the cytokinesis phase of mitosis. This results in the formation of mononucleated, lobulated, or binucleated cells.^[^ ^41^ ^]^

An endocycling cell faces at least two common issues: how to initiate endoreplication instead of mitosis, and how to alternate between the G and S phases. To address the first issue, a braking signal must be activated in the mitotic machinery without blocking the S phase. This is achieved by downregulating mitotic cyclin‐dependent kinases (M‐CDKs, including cyclin A‐CDK1 and cyclin B‐CDK1) while maintaining synthesis activity (S‐CDK, including cyclin E‐CDK2 and cyclin A‐CDK2).^[^ ^33^ ^]^ M‐CDK can be proteolytically degraded through E3 ubiquitin ligase, anaphase‐promoting complex/cyclosome (APC/C) accompanied by its coactivators Cdc20 and Cdh1 sequentially in the M and G1 phases or inhibited by a cyclin‐dependent kinase inhibitor (CKI). The oscillating expression of the transcriptional activator E2F is required in *Drosophila* models to maintain the repeated progression of G/S or G/S/truncated M phases. As in trophoblast giant cells in the mammalian placenta (Figure 2c), the programmed endocycle is orchestrated by APC/C^Cdh1^ activation, CKI p57^Kip2^ induction, and regulating the expression of different E2Fs, reaching a DNA content of up to 64N.^[^ ^40b^ ^]^

The dysregulation of cyclin‐CDK activity is also associated with PACC formation (Figure 2d).^[^ ^42^ ^]^ Amplification of *CCNE1* (encoding G1/S specific cyclin E1) facilitates polyploidization, independent of the cancer type.^[^ ^6^ ^]^ A previous study showed that cyclin E1 overexpression promotes liver‐specific polyploidization in hepatocellular adenomas through premature S‐phase entry and erroneous DNA replication.^[^ ^43^ ^]^ Zeng J. et al. recently demonstrated that this accelerated S‐phase entry, triggered by elevated cyclin E expression, induces replicative stress in cancer cells, which results in prolonged G2 arrest and final mitosis‐bypass in a p53‐p21 axis‐dependent manner.^[^ ^44^ ^]^ In G2‐arrested cells, p53 regulates the CKI p21 to suppress CDK activity. CDKs inhibit APC/C activity; as a result, APC/C^Cdh1^ is activated, creating a window for degrading the M‐CDK complex and facilitating mitotic bypass. Additionally, the expression of cyclin E inhibits p53‐dependent senescence following mitotic bypass, allowing endocycling.^[^ ^44^ ^]^ In p53‐deficient cells, damaged telomeres or DNA‐double strand breaks (DSBs) directly activate checkpoint kinase 1 (CHK1) signaling to suppress CDK activity and induce endocycling.^[^ ^45^ ^]^ Furthermore, since the altered expression of CKI can suppress M‐CDK activity and block M phase entry, accumulation of the CKI p27^kip^ has been reported to boost polyploidization and promote tumorigenesis.^[^ ^46^ ^]^

During endomitosis, cells initiate the mitotic cycle but exit mitosis without complete karyokinesis. Physiological endomitosis is exemplified by megakaryocytes (MKC), whose ploidies can reach up to 128N. Their large size is indispensable for producing anucleated platelets during blood clotting (Figure 2e). This process is facilitated by decreased actomyosin levels in the cleavage furrow required for cell separation, which arises from impaired RhoA activity.^[^ ^47^ ^]^ Microtubule motor factors, such as mitotic aurora kinases, polo‐like kinase, and APC/C signaling, are also involved in MKC endomitosis.^[^ ^48^ ^]^

Endomitosis often contributes to the failure of anti‐mitotic drugs for cancer treatment. By interfering with microtubule depolymerization or polymerization (for example, taxanes and vinca alkaloids, respectively), these classic spindle poisons disrupt proper attachment of microtubules to kinetochores and trigger the prolonged activation of the spindle assembly checkpoint (SAC). When the SAC monitors abnormalities, it brakes cell division. One key consequence of this is that two important proteins, securin and cyclin B1, are protected from APC/C degradation. Since securin facilitates the cohesion of sister chromatids and cyclin B1 governs cell cycle progression, the protection halts metaphase.^[^ ^49^ ^]^ However, mitotic arrest results not only in cell death but also in “mitotic checkpoint adaptation” and mitotic slippage (Figure 2f).^[^ ^50^ ^]^ Mechanistically, protracted SAC activation causes fluctuations in cell fate based on the balance between the accumulation of apoptotic signals and the strength of the APC/C machinery, specifically, the degradation rate of cyclin B1. Cyclin B1 degradation plays a crucial role in determining cell fate during mitosis. Insufficient activation leads to mitotic catastrophe, whereas a premature decline results in mitotic slippage. For example, the activation of JNK and Yorkie (a YAP homolog and Hippo pathway effector) in *Drosophila* imaginal epithelia induces the generation of PACCs by downregulating cyclin B.^[^ ^51^ ^]^ The knockdown of cyclin B1 also significantly increased the frequency of PACCs in breast and ovarian cancers.^[^ ^52^ ^]^ As the translocation of cyclin B1‐CDK1 to the nucleus is necessary for mitosis entry, the cytoplasmic localization of cyclin B1 may delay the G2/M transition and contribute to PACC formation.

### 2.5 Cytokinesis Failure

Cytokinesis is a complex and dynamic process consisting of several sequential steps, including division plane alignment, cleavage furrow ingression, midbody formation, and abscission. Defects in any of these steps can lead to cytokinesis failure, ultimately resulting in cellular polyploidy. Due to the major biophysical challenges involved in cytokinesis, errors in this process can occur frequently. The presence of a large amount of chromatin resulting from chromosomal non‐disjunction, as well as the occurrence of a single lagging chromosome or chromatin bridge, disturbs the completion of cytokinesis^[^ ^53^ ^]^ (Figure 2f). The primary cause of segregation barriers is merotelic chromosome attachment, where a single kinetochore is attached to the microtubules from both spindle poles. Merotelic attachment can trap chromatids in the cleavage furrow.^[^ ^53^ ^]^ While merotelic attachments can potentially induce a delay in abscission dependent on aurora B kinase,^[^ ^54^ ^]^ they can still fulfill the requirements of the SAC spindle if these attachments are left uncorrected, thereby enabling anaphase to proceed with lagging chromosomes.^[^ ^55^ ^]^ Cytokinesis failure can also result from the altered expression of proteins that govern cytokinesis. The loss of oncosuppressor genes, such as cytokinesis checkpoints, large tumor suppressor 1/2 (*LATS1/2*)^[^ ^56^ ^]^ and cell cycle regulator, breast cancer susceptibility genes 1/2 (*BRCA1/2*),^[^ ^57^ ^]^ results in defects either in the initiation of cleavage furrow ingression or in the completion of abscission, increasing the occurrence of tumor multinucleation.^[^ ^53^ ^]^

After mitotic slippage or cytokinesis failure, tetraploids must surmount the G1 restriction point to reinitiate DNA replication. The existence of a p53‐dependent “tetraploid checkpoint” is controversial; if it exists, it implies that it detects excess chromosomes or centromeres. Supporting evidence suggests that tetraploid cells with functional p53 undergo permanent G1 arrest when cytokinesis fails, inhibiting proliferation; tetraploid cells lacking p53 can replicate and divide.^[^ ^58^ ^]^ Other studies have argued against this point,^[^ ^59^ ^]^ and proposed that p53‐mediated G1 arrest after tetraploidization is most likely due to DNA damage from previous abnormal mitoses rather than the increase in ploidy itself.^[^ ^60^ ^]^ In the classic G1 DNA damage checkpoint, the p53‐p21^CIP1/WAF1^ pathway inhibits cyclin‐CDK2 complexes, which are responsible for the G1‐S transition by phosphorylating pRb to free E2Fs. Following early *TP53* mutations or E2F‐mediated G1 arrest defects in p53 wild‐type cancer, the odds of tetraploidization in cancer cells increase,^[^ ^6^ ^]^ whereas G1 arrest following tetraploidization can be overridden by cyclin D overexpression.^[^ ^61^ ^]^ Furthermore, the presence of extra centrosomes and increased cell size in tetraploid cells triggers the tumor suppressor Hippo‐YAP pathway, which involves altered small G protein signaling, including RAC1 and RHOA. This leads to the activation of the LATS2 kinase, which stabilizes p53 by counteracting its E3 ubiquitin ligase, MDM2 (attributable to the constant degradation of p53 in an unstressed state).^[^ ^62^ ^]^ p53 can also be activated by supernumerary centrosomes upon cytokinesis failure to stimulate the PIDDosome multiprotein complex, leading to MDM2 cleavage by caspase‐2.^[^ ^60^, ^63^ ^]^

## 3 Mechanisms of Depolyploidization in PACCs

Upon escaping G1 arrest, tetraploid cells enter a new round of cell division accompanied by various challenges, such as replication stress during the first S phase,^[^ ^64^ ^]^ activation of pro‐apoptotic pathways,^[^ ^65^ ^]^ induction of senescence (especially in p53 wild‐type settings), or polyploidy reversal (depolyploidization). The term “depolyploidization” was first coined by Grell in 1953 based on the observation that the polyploid ciliate *Aulocantha* cell segregated its genomes by nucleus separation.^[^ ^66^ ^]^ Owing to depolyploidization‐induced variability, depolyploidization plays a vital role in cell adaptation and cancer development under cytotoxic stress.^[^ ^67^ ^]^

### 3.1 Ploidy‐Reductive Mitosis

**Figure 3:**
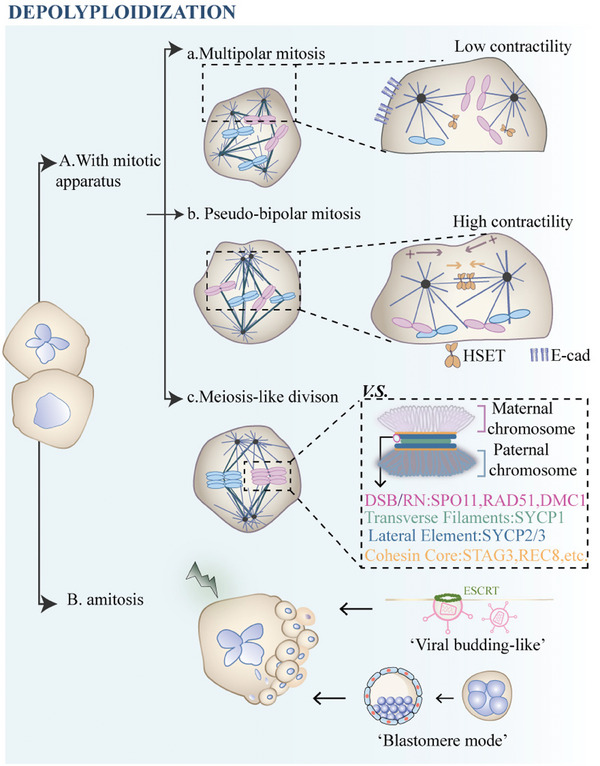
Molecular mechanisms proposed for depolyploidization of PACCs. Oncotherapy‐induced mono‐ or multinucleated PACCs undergo depolyploidization to generate tumor‐initiating daughter cells with reduced ploidy. Ploidy reductive divisions are divided into two types according to their dependence on mitotic spindles: A) mitosis‐like division, and B) asymmetric amitosis. Mitosis‐like division involves mitotic apparatus and encompasses multipolar, pseudobipolar, and meiosis‐like divisions. a) Although multipolar mitosis with supernumerary centrosomes is widely considered to cause lethal aneuploidy, non‐random multipolar segregation has been observed in some physiological cases. However, the underlying mechanisms remain largely unknown. b) Pseudo‐bipolar mitosis uses “centrosome clustering” to distribute multiple chromosomes and centrosomes, resulting in low‐grade but viable chromosomal instability. The presence of E‐cadherin prevents centrosome clustering by reducing cortical contractility, whereas loss of E‐cadherin increases contractility and allows the HSET motor to function at an optional inter‐centrosome distance. c) Meiosis‐like division involves cosegregation of diplochromosomes and reactivation of meiosis‐specific genes. B) In asymmetric amitosis, previously termed “neosis”, giant nuclei of PACCs are fragmented by nuclear budding, and the small nucleus is split off with acquired cytoplasm to form a daughter cell. ESCRT proteins are highly expressed in viral‐budding like division of PACCs. The “Blastomere mode” proposed by Liu et al. compares and unifies the giant cell cycle with embryonic development cycle. Abbreviations: E‐cad, E‐cadherin; ESCRT, an endosomal sorting complex required for transport; DSBs, double‐strand breaks; RN, recombination nodules.

Polyploid cells with supernumerary centrosomes develop multipolar mitotic spindles, causing severe chromosome missegregation (**Figure** **3**A and Table 1). Mounting evidence suggests that this can generate three or more daughter cells with lower ploidy but high levels of aneuploidy, predisposing the offspring to mitotic catastrophes or cellular demise.^[^ ^68^ ^]^ However, studies on human embryos have estimated that ≈12% of one‐cell‐stage embryos exhibit multipolar divisions.^[^ ^69^ ^]^ Frade et al. recently discovered that controlled ploidy‐reductive mitosis corrects fusion‐derived tetraploidy in early mouse embryos. In this case, a non‐random tripolar division was observed, during which the two parental chromosomes remained spatially separated.^[^ ^70^ ^]^ Although the mechanistic details for faithful multipolar mitosis remain an enigma, a study focusing on the in vivo environment might offer a new possibility that tissue architecture protects the fidelity of chromosomal segregation in an integrin‐dependent manner.^[^ ^71^ ^]^ Another study using in vivo lineage tracing revealed that hepatocyte progenies generated through ploidy‐reductive mitosis play a crucial role in polyploidy‐mediated liver regeneration.^[^ ^2b^ ^]^ Chromosome segregation during multipolar mitosis is rarely random, but reliable. As human polyploid hepatocytes are mainly binuclear tetraploids (2 × 2N),^[^ ^72^ ^]^ one possible mechanism is that the genetic material in each nucleus of parental hepatocytes stays with each other during ploidy reduction in mitosis. Although ploidy reduction efficiently contributes to the reconstruction of the injured liver, multiple rounds of ploidy reduction from normal polyploid hepatocytes initiate an early step in tumorigenesis.^[^ ^2c^ ^]^

Given that faithful multipolar mitosis remains limited, the highly aneuploid offspring produced by error‐prone ploidy reduction are mostly non‐viable. A process named “centrosome clustering” was observed in PACCs, where amplified centrosomes were clustered at two mitotic poles, and pseudo‐bipolar spindles were formed to segregate chromosomes with higher fidelity.^[^ ^73^ ^]^ It is notable that mammalian tetraploid cells capable of centrosome clustering exhibit higher rates of merotelic attachment and lagging chromosomes than diploid cells.^[^ ^74^ ^]^ As the SAC poorly senses merotelic attachment, PACCs deploy centrosome clustering to avoid disastrous outcomes by allowing low‐grade but viable chromosome instability (Figure 3a,b). Rhys et al. proposed a two‐step model for ultimate centrosome clustering containing a slow search‐and‐capture phase caused by actomyosin contractility and a subsequent fast‐motorized phase mediated by HSET, a microtubule kinesin motor encoded by *KIFC1*. Since E‐cadherin represses the first rate‐determining step in this process, the loss of E‐cadherin could provide elevated tolerance to centrosome amplification in cancers and serve as an adaptation mechanism for PACC division.^[^ ^75^ ^]^ In addition, a significant enrichment of *PPPR21A* mutations has been observed in polyploid tumors. *PPPR21A* mutations cause loss of function of phosphatase 2A (PP2A‐Aα) in mitosis and promote centrosome clustering.^[^ ^8^, ^76^ ^]^

In addition to ploidy‐reductive mitosis, meiosis‐like reductive division has been reported during the depolyploidization of PACCs (Figure 3c).^[^ ^77^ ^]^ In a Notch‐induced *Drosophila* tumor model, genes involved in meiotic recombination of homologous chromosomes were upregulated and indispensable in the tumor cells to undergo ploidy‐reduction.^[^ ^78^ ^]^ During the anaphase of radiation‐induced PACCs, an increase in Rec8 expression was observed to enhance sister centromeric cohesion and the co‐segregation of diplochromosomes (pairing chromosomes with connected chromatids), which exhibit morphological similarities to meiotic prophase I.^[^ ^79^ ^]^ Other meiosis‐specific genes, the so‐called cancer testes‐associated antigens, including the meiosis‐specific endonuclease SPO11, mitotic/meiotic recombinases RAD51 and DMC1 in early recombination nodules, and synaptonemal‐complex proteins SYCP1/2/3 are also required for the onset of depolyploidization.^[^ ^77^, ^80^ ^]^ All these data indicate that meiotic‐like functions may provide a structural and molecular basis for polyploidy reversibility in tumor cells. Hence, it is plausible that the involvement of meiosis‐like programs in depolyploidization increases the heterogeneity of tumor cells, as in gametes.

### 3.2 Amitosis

An alternative ploidy reduction process devoid of mitotic spindles, called amitosis, has been described in many cases from primitive ciliates to mammals (Figure 3B and Table 1). Lucchetta and Ohlstein highlighted amitosis as an unexpected mechanism for stem cell self‐renewal in the *Drosophila* intestine, during which polyploid enterocytes undergo nuclear invagination to resolve binucleate cells into two fully functional stem cells.^[^ ^81^ ^]^ The nuclear budding of para‐diploid progeny has also been documented in the depolyploidization of PACCs following cytotoxic therapy.^[^ ^67^, ^82^ ^]^ During this process, small nuclei bud from the giant mother nucleus and sprout from the surface of polyploid cells via intracellular cytokinesis, separating tumor‐initiating daughter cells with a much lower ploidy. Without the breakdown of nuclear envelopes or the involvement of the spindle apparatus, this genome reduction process is probably orchestrated by intranuclear chromosome segregation.^[^ ^83^ ^]^ However, the mechanism by which PACCs accurately distribute complex chromosomes evenly to the budded daughter cells remains unclear. Our single‐cell sequencing results showed that although some newly generated cells may still exhibit chromosomal abnormalities, they actively repair and adjust to a chromosomal state similar to that of the parent cell within several cell division cycles.^[^ ^13^ ^]^

Nuclear buds are entangled in cytoplasmic blebs to achieve asymmetric cell budding. These cellular protrusions were recently reported to increase tumor invasiveness by activating pro‐survival NRAS‐ERK‐PI3K signaling in a melanoma model.^[^ ^84^ ^]^ An interesting parallel was observed in the rapid evolution of the bacterium *Escherichia coli*, where *E.coli* filaments were shown to bud off progeny with enhanced resistance through delicate recombination between chromosomes.^[^ ^85^ ^]^ In yeast, budding is a mode of asexual division that polarizes the actin cytoskeleton and organizes membrane trafficking. The major player in this cellular polarization process is the small GTPase Cdc42, which also affects budding daughter cells from PACCs.^[^ ^86^ ^]^ Our recent work revealed that the endosomal sorting complex required for transport (ESCRT) proteins are highly involved in the budding of PACCs, strikingly reminiscent of ESCRT‐dependent viral vesicle maturation and budding from the host plasma membrane (Figure 3B).^[^ ^13^ ^]^ Furthermore, radiation‐induced PACCs release nucleic acids into the cytoplasm, which activate antiviral signaling through the type I interferon pathway. The exposure of early PACCs to additional interferon enhances host antiviral effects and prevents tumor relapse through PACC budding, which has promising therapeutic implications.^[^ ^13^ ^]^

Senescence is an important consideration for PACC formation and ensuing asymmetric division. Replicated senescent cells continuously exit the cell cycle and enter permanent growth arrest, whereas therapy‐induced accelerated senescence can initiate a prolonged but reversible cell cycle arrest.^[^ ^87^ ^]^ Early in 2004, Sundaram and Rajaraman proposed that accelerated senescence coupled with giant polyaneuploidy in cancer cells could allow them to undergo nuclear budding and give rise to viable Raju cells, whereas the polyploid mother cells ultimately die.^[^ ^88^ ^]^ They named this ploidy‐reductive paradigm as “neosis”. Abbadie et al. observed a spontaneous transformation from senescence to neoplasia via *a* two‐step budding‐off cytokinesis using human mammary epithelial and epidermal keratinocytes.^[^ ^89^ ^]^ In this transformation, the p16‐RB pathway responds at the outset and mediates initial budding, which occurs at a low frequency. Following senescence, cells enter a second growth plateau, during which the activation of p53‐p21 or p16‐RB leads to another budding event accompanied by the acquisition of tumorigenic properties.^[^ ^90^ ^]^ Oxidative stress‐induced accumulation of single‐strand breaks appears to be at the center of all these events. Furthermore, NF‐κB‐mediated secretome changes are associated with the secretory phenotype of senescence, stimulating the development of premalignant cells and acquiring tumor resistance.^[^ ^91^ ^]^ Collectively, these studies revealed that senescent PACCs can change their fate via unusual amitosis, indicating the potential plasticity of senescent PACCs.

**Figure 4:**
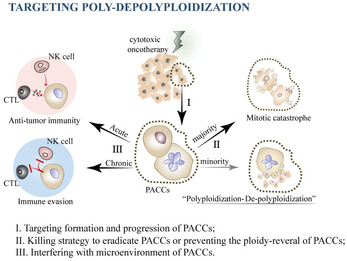
Strategies to target “poly‐depolyploidization”. Three approaches are discussed in the text and shown in **Table** **2**.

**Table 2:** Targets in the prevention of ploidy variation.

Drugs		Indication and clinical status	Outcome	References
targeting the formation and progression of PACCs				
KIF18A inhibitor	AM‐5308, AM‐9022 AM‐0277, AM‐1882	p53‐mutant high‐grade serous ovarian cancer, triple‐negative breast cancer with CIN features, preclinical	Tumor regression and favorable tolerability in mice	[114]
	VSL‐1488	Advanced solid tumor, phase I/II	Ongoing recruitment	NCT05902988
	AMG650	High‐grade serous ovarian cancer and advanced solid tumor, phase I	Ongoing recruitment	NCT06084416, NCT04293094
	GH2616	Clinical trial application was approved by NMPA, preclinical	Selectively inhibits the growth of cells with p53‐ mutant	[115]
CDK2 inhibitor	Mifepristone	Non‐small cell lung cancer, preclinical	Impairs the repopulation of tumor cells following cisplatin therapy.	[99]
Autophagy inhibition	Hydroxychloroquine or BML‐275	Nasopharyngeal carcinoma, preclinical	Pretreatment before chemotherapy maximizes the therapeutic index	[11f]
inducing apoptosis of PACCs				
Senolytics	ABT263	Melanoma and lung cancer, preclinical	Selective clearance of senescent cells	[101b]
preventing depolyploidization of PACCs				
HSET inhibitor	CW069	Prostate cancer, preclinical	Induces apoptosis and overcomes resistance to docetaxel	[103b]
ASAH1 inhibitor	Tamoxifen	Prostate cancer, glioblastoma, and melanoma cells, preclinical	Reduces the repopulation of tumor cells after irradiation	[103b]
intervening in the tumor microenvironment				
STING inhibitor	C‐176, H‐151	Melanoma, breast and colorectal cancers, preclinical	Attenuates CIN high tumor progression	[112]

NMPA: National Medical Products Administration

Recent studies have reported that senescent cells undergo epigenetic reprogramming to generate tumor‐initiating cells.^[^ ^92^ ^]^ It is not surprising that PACCs acquire stemness to mediate the initiation, maintenance, and recurrence of malignancy. Several laboratories have reported the presence of stemness reminiscent of embryos in PACCs, supporting this viewpoint.^[^ ^82^, ^93^ ^]^ The activation of core embryonic stem cell markers (Oct4, Nanog, and Sox2) was detected in PACCs in a spatiotemporally dependent manner, which was found to form highly tumorigenic spheroids in vivo capable of differentiating into major germline cell layers. Furthermore, both PACCs and their progeny express classic stem cell markers (e.g., CD44) and exhibit resistance to chemotherapy. Based on these findings, Liu et al. proposed that PACCs represent somatic equivalents of blastomeres (Figure 3B).^[^ ^93^ ^]^ During the self‐renewal phase, stressor‐induced PACCs undergo cleavage, reminiscent of blastomeres, compaction, and morula formation, before developing into an inner stem‐cell mass resembling a blastocyst. The subsequent differentiation of these cells can initiate tumor relapse. In line with this hypothesis, Diaz‐Carballo et al. observed that PACCs generate daughter cells with partial stemness and thus described PACCs as “pregnant cancer cells”.^[^ ^94^ ^]^ Our results reveal that radiation‐induced PACCs share similarities with cancer stem cells in terms of their association with radioresistance and cancer repopulation. However, our study suggests that PACCs with severe DSBs are not enriched in conventional stemness signatures. Instead, they enter a diapause‐like state characterized by slow cycling and reduced metabolism, enabling them to endure harsh conditions and provide better support for regeneration.^[^ ^13^ ^]^ Radiation‐induced PACCs use viral budding‐like division to re‐propagate the tumor rather than the asymmetric self‐renewal of stem cells. Overall, PACCs induced by cytotoxic therapy act as bad seeds for cancer relapse, displaying varying degrees of stemness depending on the type of DNA damage inflicted.

## 4 Therapeutic Targeting of “Poly‐depolyploidization”

Polyploidization confers resistance to therapy‐induced cell death in cancer cells. Some PACCs recover following de‐polyploidization, giving rise to extensive para‐diploid progeny with a robust ability to proliferate. This stepwise transformation endows PACCs with a selective advantage in surviving cytotoxic stressors, thus presenting the need for different treatment strategies at different stages. Here, we propose possible approaches aimed at (i) targeting the formation and progression of PACCs, (ii) preventing the depolyploidization of PACCs or inducing their apoptosis, and (iii) intervening in the tumor microenvironment that supports PACCs (**Figure** **4** and **Table** **2**).

Polyploidization imposes adaptations that create distinct genetic and phenotypic differences between somatic diploid cells and PACCs. This divergence offers a valuable opportunity for therapeutic intervention, termed “ploidy‐specific lethality”. PACCs exhibit heightened reliance on SAC signaling, DNA replication factors, and proteasomes compared to diploid cells.^[^ ^8a^ ^]^ Notably, the mitotic kinesin protein KIF18A, plays a crucial role in PACC survival. While the absence of KIF18A in diploid cells may have little impact on proper chromosome segregation during mitosis, PACCs become more susceptible to disturbances in microtubule dynamics caused by the loss of KIF18A function. This results in severe mitotic errors, which ultimately compromise cell viability.^[^ ^8^, ^95^ ^]^ Several KIF18A inhibitors (such as AM‐5308, AM‐9022, AM‐0277, and AM‐1882) have been found to have potent anti‐tumor effects in mouse models, leading to tumor regression in human models of high‐grade serous ovarian cancer (HGSOC) and triple‐negative breast cancer.^[^ ^96^ ^]^ Until now, only three small molecule compounds targeting KIF18A have entered clinical research: VLS‐1488 in phase 1/2 trials for solid tumors (NCT05902988), AMG650 in Phase I trials for solid tumors (NCT04293094) and HGSOC (NCT06084416), and GH2616 receiving approval for clinical trials. Moreover, toward PACCs with excessive centrosomes, CDK2 inhibitors exhibit selective lethality and spare normal cells with two centrosomes.^[^ ^97^ ^]^ Mifepristone, a well‐known anti‐progestin steroid, is a cytostatic compound targeting CDK2.^[^ ^98^ ^]^ Mifepristone exposure greatly impairs the escape of PACCs from DNA damage caused by cisplatin, as well as the action of poly (ADP‐ribose) polymerase inhibitors (PARPi), and is effective in halting tumor progression in non‐small cell lung cancer (NSCLC) and HGSOC.^[^ ^15^, ^99^ ^]^

The formation of PACCs also involves exploitation of specific vulnerabilities. Taking advantage of dependence on the AMPK‐mTOR mediated mitophagy pathway observed in chemotherapy‐induced nasopharyngeal carcinoma PACCs, autophagy inhibition using hydroxychloroquine or BML‐275 prior to chemotherapy prevented PACC formation and mitigated metastasis.^[^ ^11f^ ^]^ Our previous work utilized single‐cell RNA sequencing to reveal unique gene expression profiles in radiation‐induced PACCs, one of which is related to polyploidization and marked by *CST3*.^[^ ^13^ ^]^ *CST3* encodes cystatin‐C, a cytokine suggested as a potential early marker of kidney injury that has demonstrated protective effects in damaged cardiomyocytes. Notably, the depletion of CST3 produces a synergistic effect with radiation to eliminate PACCs and impede tumor repopulation following radiotherapy.

Most PACCs tend to undergo cell cycle arrest and eventual cell death, whereas a small fraction can undergo depolyploidization to revert to a diploid state. Thus, inducing the senescence and the subsequent demise of PACCs offers a promising approach. Senolytics, which selectively induce apoptosis in senescent cells, may also be used as a translational strategy.^[^ ^100^ ^]^ Combined with cytotoxic treatment, several senolytics, such as BCL‐2 family inhibitors (Navitoclax, ABT263) and kinase inhibitors (dasatinib), have yielded synergistic anticancer effects in preclinical and early clinical studies.^[^ ^101^ ^]^

Regarding the reversibility of polyaneuploidy, targeting transformation‐driven factors may provide alternative therapeutic benefits. An initial strategy is to de‐cluster extra centrosomes and render PACCs susceptible to detrimental multipolar mitosis. The microtubule motor HSET is an attractive target whose inhibition potentially eradicates damaged PACCs with extra centrosomes without affecting normal cells.^[^ ^102^ ^]^ HSET inhibitors, including CW069^[^ ^103^ ^]^ and AZ82,^[^ ^104^ ^]^ have been shown to markedly preclude centrosome clustering, decrease pseudo‐bipolar division, and increase apoptosis of cancer cells. Another potential therapeutic target is amitosis. ASAH1, or acid ceramidase, converts pro‐apoptotic ceramide to sphingosine, which is then phosphorylated to produce anti‐apoptotic sphingosine‐1‐phosphate (S1P). As a rheostat, ASAH1 ultimately determines whether a cell will become a tumor promoter or suppressor.^[^ ^105^ ^]^ By altering sphingolipid metabolism, the inhibition of ASAH1 shows promise as a potential therapeutic strategy in combination with cytotoxic treatments to suppress the primitive, cleavage‐like division of PACCs.^[^ ^106^ ^]^ Although specific ASAH1 inhibitors are not yet available in clinical trials, tamoxifen, an estrogen receptor (ER) antagonist, has been shown to decrease the number of PACC offspring by repressing ASAH1 expression rather than through ER signaling.^[^ ^106b^ ^]^ Likewise, our sequencing data suggested that certain genes are involved in the budding of irradiation‐induced PACCs, of which *SNCG* is a representative gene. *SNCG* encodes the protein synuclein gamma, an amyloidogenic protein induced by stress. Inhibiting SNCG expression has been shown to eliminate PACC budding in preclinical colorectal cancer models.^[^ ^13^ ^]^

Another important consideration is the role of the immune system in the progression of PACC‐related diseases. Hyperploid cancer cells that suffer constitutive endoplasmic reticulum stress aberrantly express calreticulin on their cell surface, making them immunogenic and inducing cytotoxic T lymphocytes‐mediated immune responses.^[^ ^107^ ^]^ The presence of extra centrosomes during polyploidization triggers PIDDosome pathway activation, inducing a pro‐inflammatory phenotype in macrophages and increasing their vulnerability to natural killer (NK) cell‐mediated attacks.^[^ ^96^ ^]^ Furthermore, chromosome segregation errors in PACCs initiate innate immune signaling by forming micronuclei and exposing double‐stranded DNA to the cytosol, which activates the antiviral cGAS‐STING pathway and promotes type I interferon production.^[^ ^108^ ^]^ Indeed, various immune effectors, including B cells, NK cells, NKT cells, and T cells, along with innate immune responses involving the interleukin‐1 receptor and Toll‐like receptor systems, participate in the immune clearance of hyperploid cancer cells.^[^ ^109^ ^]^ However, a recent sequencing study using pan‐cancer data reported a negative enrichment of tumor‐infiltrating leukocytes and inflammatory processes in polyploid tumors.^[^ ^8a^ ^]^ Similarly, highly aneuploid tumors exhibit a loss of immune signatures, as aneuploidy disrupts local immunity through an unfolded protein response and impairs immune surveillance.^[^ ^108^, ^110^ ^]^ Consistent with this immunosuppressive environment, the slow‐cycling state facilitates immune evasion by reducing the expression of cell‐surface‐innate immune sensors.^[^ ^111^ ^]^ PACCs could also adapt to chronic CIN by rewiring cGAS‐STING signaling with endoplasmic reticulum (EnR) stress signaling, allowing them to evade inflammation caused by type I interferon while benefiting from the immunosuppressive properties of EnR stress signaling.^[^ ^108^, ^112^ ^]^ Inhibiting cGAS‐STING signaling, such as via treatment with a STING inhibitor, could potentially prevent CIN‐related chronic inflammation and offer therapeutic opportunities for tumors with high levels of PACCs.^[^ ^112^ ^]^

A thorough analysis of the correlation between PACCs and the immune system, particularly by examining on‐treatment biopsies from patients with MRD, is of utmost importance to gain a comprehensive understanding of the intricate role played by the tumor microenvironment. This aspect, characterized by its complexity, presents a considerable challenge when investigated solely through in vitro or in vivo immunocompromised mouse models. Moreover, little is known about whether PACCs persist and undergo depolyploidization following immune checkpoint blockade (ICB). Importantly, polyploid tumors appear to exhibit a better response to ICB,^[^ ^8a^ ^]^ although the mechanism underlying this phenomenon is presently unknown. Tumors with high levels of aneuploidy exhibit enhanced responsiveness to a concurrent combination of ablative radiotherapy and ICB, leading to improved tumor responses and overall survival rates. This benefit was not observed in patients with few aneuploid tumors.^[^ ^113^ ^]^

### 4.1 Concluding Remarks

Despite the growing interest in the role of PACCs in cancer, several critical questions remain unanswered. In stress‐induced polyploidization, what determines the choice of cancer cells between cell cycle‐independent cell fusion and cell cycle‐dependent endoreplication remains an important research question. Moreover, knowing if polyploidization is associated with the subsequent depolyploidization and whether specific mechanisms favor budding‐off amitosis instead of mitosis or meiosis‐like division remains to be elucidated. Finally, it is also important to uncover if amitotic PACCs reorganize and reassemble multiple copies of chromosomes and centrosomes to assign them to para‐diploid progeny. High‐resolution analytical technologies, such as single‐cell multi‐omics analysis, are required to determine if complex genomic reorganization is involved in the phenotypic switching of PACCs, especially in a highly heterogeneous and dynamic context. It might also be the key to utilizing organoids or patient‐derived tumor models, ideally combined with CRISPR/Cas9‐mediated lineage tracing technology, to track the in vivo dynamics of the ploidy in a spatiotemporal manner.

In conclusion, a small fraction of residual cancer cells survive therapeutic challenges by undergoing the “poly‐depolyploidization” process, which may be an important mechanism underlying cancer resistance and relapse after cytotoxic therapy. Therefore, it would be a transformative clinical advancement to develop treatments targeting this mechanism to improve the outcomes in patients with various malignancies.

## Conflict of Interest

The authors declare no conflicts of interest.
